# Effects of Biochar Addition and Nitrogen Application Rate on Soil Properties and Agronomic Nitrogen Use Efficiency in Artificial Grasslands

**DOI:** 10.3390/plants15132097

**Published:** 2026-07-06

**Authors:** Wenhao Wang, Asitaiken Julihaiti, Helong Yang, Xin Wang, Kejian Lin, Zhi Xing, Lingqi Kong

**Affiliations:** 1 College of Grassland Science, Xinjiang Agricultural University, Urumqi 830052, China; 18599460192@163.com (W.W.); astekin77@163.com (A.J.); 1540361482@163.com (X.W.); 2 Xinjiang Key Laboratory of Grassland Resources and Ecology, Urumqi 830052, China; 3 Key Laboratory of Grassland Resources and Ecology of Western Arid Region, Ministry of Education, Urumqi 830052, China; 4 Institute of Grassland Research, Chinese Academy of Agricultural Sciences, Hohhot 010010, China; linkejian@caas.cn (K.L.); xingzhi@caas.cn (Z.X.); konglingqi@caas.cn (L.K.)

**Keywords:** biochar, N rate reduction, agronomic nitrogen use efficiency, hay yield, soil moisture, available nitrogen, plant community diversity, artificial grassland, structural equation modeling

## Abstract

In modern livestock production, a reliable supply of high-quality forage is essential for sustaining animal productivity and product quality. Although nitrogen (N) fertilization can promote forage growth, excessive N inputs often result in low agronomic nitrogen use efficiency (NAUE) and increased environmental risks. Biochar, owing to its porous structure, high specific surface area, and physicochemical stability, can improve soil physical properties, enhance water and nutrient retention, and regulate soil N availability. However, the mechanisms by which biochar combined with reduced N rate fertilization affects NAUE in artificial grasslands remain insufficiently quantified. A two-year field experiment was conducted at the Grassland Science Experimental Station of Xinjiang Agricultural University on the northern slope of the Tianshan Mountains, Xinjiang, China. Eight treatments were established using a factorial design with two biochar rates (0 and 20 t·ha^−1^; B0 and B20) and four N application rates (0, 75, 150, and 225 kg·ha^−1^; N0, N75, N150, and N225). Results showed that biochar application significantly decreased soil bulk density and increased soil water content and electrical conductivity. It also elevated soil total carbon, total nitrogen, total phosphorus, NH_4_^+^–N, and NO_3_^−^–N concentrations, with B20N150 exhibiting the highest overall nutrient status. Plant community diversity indices did not differ significantly among treatments (*p* > 0.05), though B20 slightly enhanced Shannon–Wiener and Simpson indices under N0 and N75. Moderate N application significantly increased hay yield, whereas the highest N rate (225 kg·ha^−1^) did not further improve yield and reduced NAUE. Biochar combined with N75 or N150 improved NAUE, and B20N150 achieved the best balance of high hay yield and high NAUE. Structural equation modeling revealed that soil water content (path coefficient = 0.45), NH_4_^+^–N (0.27), and plant community diversity (0.20) were key positive drivers of NAUE, with biochar exerting indirect effects primarily via improving soil water and available N. Collectively, applying 20 t·ha^−1^ biochar with 150 kg·ha^−1^ N (B20N150) is recommended as an optimal strategy for N rate reduction and NAUE enhancement in artificial grasslands of arid and semiarid regions.

## 1. Introduction

Grasslands constitute a fundamental component of terrestrial ecosystems and serve as a critical natural resource underpinning livestock production and ecological security [1]. As the material basis of pastoral animal husbandry, forage grasses directly determine livestock feeding standards and the overall economic returns of grassland-based production systems through their yield and nutritional quality [2]. Artificial (sown) grasslands, established and managed through agronomic practices including seeding, fertilization, irrigation, and mowing, are designed to provide high-yielding, high-quality, and stable forage supplies, thereby compensating for the limited productivity of natural grasslands and meeting the increasing demand for livestock feed [3]. However, prolonged utilization of artificial grasslands frequently leads to soil fertility depletion, simplification of community structure, and deterioration of forage yield and nutritional quality, primarily driven by intensive harvesting regimes, continuous nutrient removal, and suboptimal management practices [4,5]. Consequently, developing scientifically sound nutrient management strategies that sustain grassland productivity while enhancing resource use efficiency represents a pressing challenge for the sustainable development of grassland agriculture.

Nitrogen (N) is one of the essential macronutrients required for forage growth and development and constitutes a key limiting factor governing the productivity of artificial grasslands [6]. Appropriate N fertilizer application promotes root and shoot growth, enhances photosynthetic capacity and assimilate accumulation, and thereby increases plant height, tiller number, aboveground biomass, and ultimately forage yield [7,8]. Nevertheless, long-term or excessive N fertilization not only increases N losses and reduces N use efficiency but may also trigger a cascade of adverse environmental consequences, including soil acidification, eutrophication of water bodies, and elevated greenhouse gas emissions. With the deepening implementation of green agricultural development concepts, reducing irrational chemical fertilizer inputs and improving fertilizer use efficiency have become central priorities in the governance of agricultural non-point source pollution and the sustainable management of grassland ecosystems. In 2019, a national policy document in China explicitly stated that efforts should be made to “strengthen the control of agricultural non-point source pollution, implement fertilizer and pesticide reduction actions, and achieve negative growth in chemical fertilizer and pesticide use” [9]. Therefore, exploring effective approaches for N reduction and efficiency enhancement in artificial grasslands—maintaining or improving forage yield while decreasing N inputs—is of considerable significance for the green and efficient development of grassland-based animal husbandry.

Biochar is a carbon-rich material produced through the pyrolysis of biomass under oxygen-limited conditions, characterized by a well-developed pore structure, high specific surface area, strong physicochemical stability, and pronounced adsorption capacity [10,11]. As a novel amendment that integrates carbon sequestration, emission reduction, and soil amelioration functions, biochar has attracted increasing research attention in agricultural and grassland ecosystem studies in recent years. Previous investigations have demonstrated that biochar incorporation into soil can improve soil physical structure by reducing bulk density, increasing porosity, and enhancing water-holding capacity and aeration. Simultaneously, biochar can regulate soil pH, strengthen cation exchange capacity and nutrient adsorption, thereby mitigating nutrient leaching risks and improving soil fertility retention [12,13]. Moreover, biochar itself contains appreciable quantities of organic carbon along with mineral elements such as N, phosphorus (P), and potassium (K), which can be slowly released into the soil to supplement plant nutrient demands [14,15,16]. These slow-release and adsorptive properties contribute to reducing chemical fertilizer application rates, improving fertilizer use efficiency, and lowering the risk of agricultural non-point source pollution [17,18]. Accordingly, the combined application of biochar and N fertilizer may represent a promising management strategy for achieving simultaneous N reduction, yield maintenance, and efficiency enhancement in artificial grasslands.

The combined application of biochar and N fertilizer has recently been recognized as a promising strategy for improving soil fertility, sustaining crop productivity, and enhancing N use efficiency. Numerous studies have shown that biochar can interact with applied N fertilizer by altering soil physical structure, increasing water retention, improving cation exchange capacity, and providing abundant adsorption sites for inorganic N, thereby reducing N loss through leaching, volatilization, and gaseous emissions [19,20,21,22]. Meta-analyses have further confirmed that biochar amendments generally increase crop yield and fertilizer use efficiency, although the magnitude of these effects depends strongly on soil type, biochar properties, application rate, climatic conditions, and fertilizer management [23,24,25]. In field experiments, biochar combined with N fertilizer has been reported to increase soil organic carbon, available N, crop biomass, and yield in maize, wheat, and rice systems, while also improving N retention and reducing environmental risks associated with excessive N inputs [26,27,28]. For example, Zhang et al. found that biochar amendment combined with N fertilization improved maize yield and reduced greenhouse gas emissions in a calcareous soil, suggesting that biochar can enhance the agronomic and environmental performance of N fertilizer use [26]. Similarly, studies in paddy soils demonstrated that biochar incorporation altered soil N transformation processes, improved soil fertility, and contributed to higher crop productivity under N fertilization [27,28]. However, most existing studies have focused on cropland systems, whereas evidence from artificial grasslands remains scarce. In arid and semiarid artificial grasslands, where soil water limitation, nutrient depletion, and low N use efficiency often occur simultaneously, the mechanisms by which biochar and reduced N fertilizer jointly regulate soil moisture, available N supply, forage yield, and agronomic N use efficiency remain insufficiently understood. Therefore, further field-based studies are needed to clarify whether biochar can serve as an effective amendment for achieving N reduction and efficiency enhancement in artificial grassland ecosystems.

Based on the foregoing considerations, we hypothesized that: (i) moderate N fertilizer addition increases forage yield and agronomic N use efficiency (NAUE), whereas excessive N application diminishes NAUE; (ii) biochar amendment improves soil structure, enhances soil water content and available nutrient concentrations, thereby optimizing the growing environment for forage species; (iii) the co-application of biochar with moderate N fertilizer exerts synergistic effects, enabling the maintenance or improvement of forage yield with reduced N inputs while enhancing NAUE; and (iv) soil moisture, available N, and plant community diversity serve as important mediating factors driving variations in NAUE.

To test these hypotheses, the present study employed a field-based controlled experiment in an artificial grassland, with treatments comprising different biochar application rates and N fertilizer levels. Soil bulk density, soil water content, soil ammonium N, plant community diversity, forage yield, and NAUE were measured. Data were analyzed using analysis of variance (ANOVA), correlation analysis, principal component analysis (PCA), and structural equation modeling (SEM), implemented through Excel, SPSS, and R software platforms. The overarching objective was to systematically elucidate the effects of biochar and N fertilizer co-application on the soil–plant system and N use efficiency in artificial grasslands, and to determine whether biochar can improve soil conditions, sustain forage productivity, and enhance NAUE under reduced N fertilization regimes, thereby providing a theoretical foundation for N-efficient and sustainable management of artificial grasslands.

## 2. Results

### 2.1. Responses of Soil Physicochemical Properties to Nitrogen Rate and Biochar

#### 2.1.1. Soil Bulk Density and Water Content

Repeated-measures ANOVA indicated that biochar and year exerted highly significant effects on soil bulk density (*p* < 0.01), whereas N fertilizer and the biochar × N fertilizer interaction did not significantly affect this parameter (Table 1). Soil water content was significantly influenced by biochar, N fertilizer, and year (all *p* < 0.01), but the biochar × N fertilizer interaction was not significant (Table 1).

**Figure 1:**
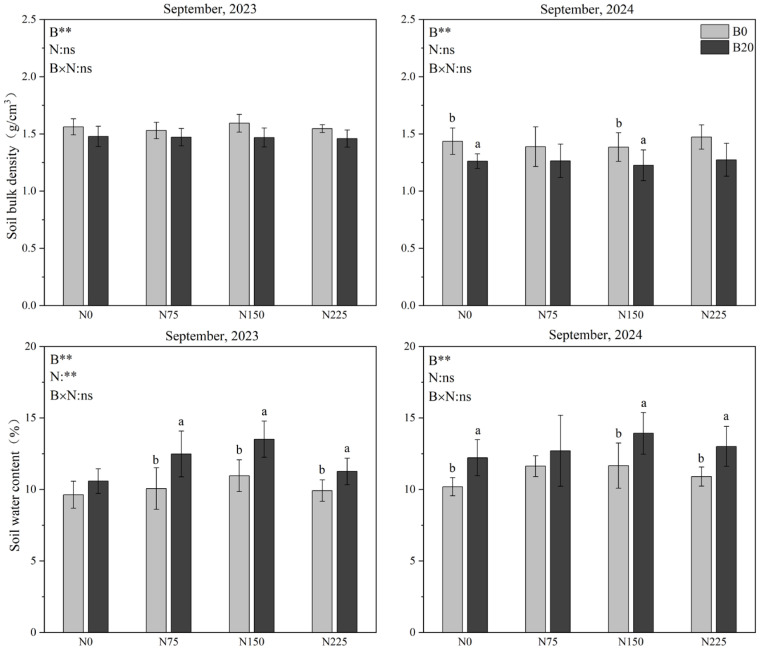
Effects of nitrogen rate and biochar addition on soil bulk density and water content. Note: N0, N75, N150, and N225 indicate nitrogen application rates of 0, 75, 150, and 225 kg N ha^−1^, respectively; B0 and B20 indicate biochar application rates of 0 and 20 t ha^−1^, respectively. Different uppercase letters indicate significant differences among nitrogen application rates under the same biochar level, whereas different lowercase letters indicate significant differences between biochar levels within the same nitrogen application rate at *p* < 0.05. The absence of letters indicates no significant difference (*p* > 0.05). Error bars represent standard errors of the mean (n = 5). B, biochar; N, nitrogen application rate; B × N, interaction between biochar and nitrogen application rate; ns, not significant. ** *p* < 0.01. The same notation applies to subsequent figures.

As shown in Figure 1, soil bulk density was consistently lower in biochar-amended (B20) plots compared with unamended (B0) plots across both years. In September 2023, differences in bulk density among treatments were relatively small. By September 2024, the bulk density-reducing effect of biochar became more pronounced, with B20 significantly lower than B0 under the N0 and N150 levels (*p* < 0.05). No consistent significant differences in bulk density were observed among N fertilizer levels.

Regarding soil water content, biochar amendment generally increased SWC across both years. In September 2023, B20 significantly increased SWC compared with B0 under the N75, N150, and N225 levels (*p* < 0.05). In September 2024, B20 significantly increased SWC under the N0, N150, and N225 levels (*p* < 0.05). N fertilizer application also influenced SWC to some extent, although the response patterns varied between years. Overall, the positive effect of biochar on SWC was consistent across both years, whereas the bulk density reduction effect was more evident in the second year.

#### 2.1.2. Soil pH and Electrical Conductivity

**Figure 2:**
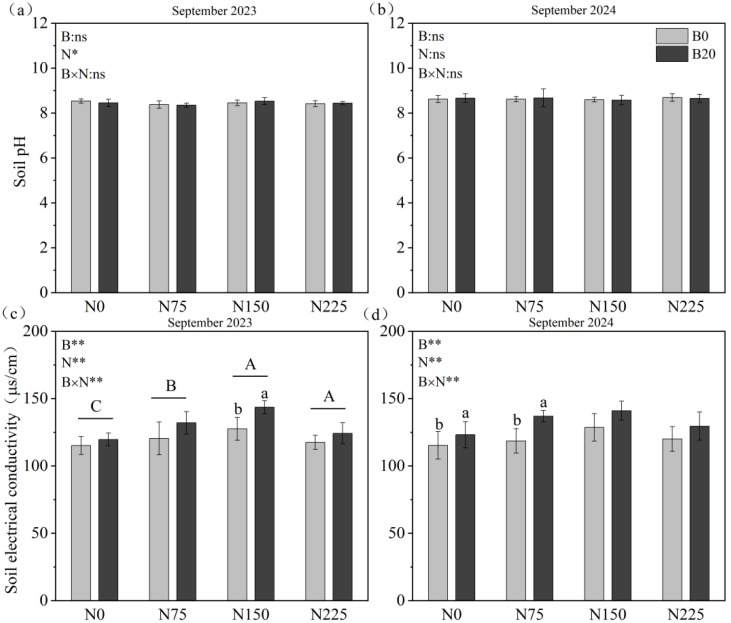
Effects of nitrogen rate and biochar addition on soil pH and electrical conductivity. (**a**) Soil pH in September 2023; (**b**) Soil electrical conductivity in September 2023; (**c**) Soil pH in September 2024; (**d**) Soil electrical conductivity in September 2024.Note: N0, N75, N150, and N225 indicate nitrogen application rates of 0, 75, 150, and 225 kg N ha^−1^, respectively; B0 and B20 indicate biochar application rates of 0 and 20 t ha^−1^, respectively. Different uppercase letters indicate significant differences among nitrogen application rates under the same biochar level, whereas different lowercase letters indicate significant differences between biochar levels within the same nitrogen application rate at *p* < 0.05. The absence of letters indicates no significant difference (*p* > 0.05). Error bars represent standard errors of the mean (*n* = 5). B, biochar; N, nitrogen application rate; B × N, interaction between biochar and nitrogen application rate; ns, not significant. * *p* < 0.05; ** *p* < 0.01. The same notation applies to subsequent figures.

As illustrated in Figure 2, soil pH exhibited minimal variation across treatments, with no consistent significant differences detected among treatment combinations. In contrast, soil electrical conductivity (EC) showed a more pronounced response to fertilization treatments. In September 2023, EC was significantly higher under N150 and N225 compared with N0 (*p* < 0.05); at the N150 level, B20 significantly increased EC relative to B0 (*p* < 0.05). In September 2024, B20 significantly increased EC compared with B0 under the N0 and N75 levels (*p* < 0.05). Overall, both biochar and N fertilizer application increased soil EC, although the magnitude of the response varied across years and N fertilizer levels.

### 2.2. Responses of Soil Nutrients to Nitrogen Rate and Biochar

#### 2.2.1. Soil Total Carbon, Total Nitrogen, and Total Phosphorus

**Figure 3:**
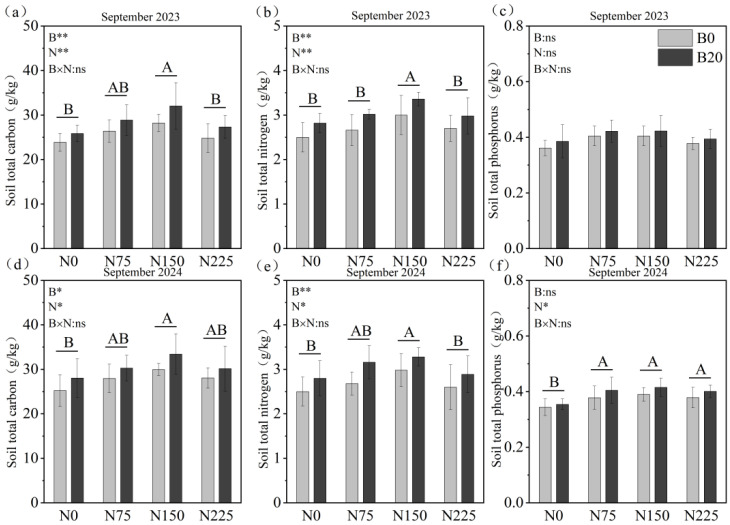
Effects of nitrogen rate and biochar addition on soil total carbon, total nitrogen, and total phosphorus concentrations.(**a**) Soil total carbon in September 2023; (**b**) Soil total nitrogen in September 2023; (**c**) Soil total phosphorus in September 2023; (**d**) Soil total carbon in September 2024; (**e**) Soil total nitrogen in September 2024; (**f**) Soil total phosphorus in September 2024 Note: N0, N75, N150, and N225 indicate nitrogen application rates of 0, 75, 150, and 225 kg N ha^−1^, respectively; B0 and B20 indicate biochar application rates of 0 and 20 t ha^−1^, respectively. Different uppercase letters indicate significant differences among nitrogen application rates under the same biochar level, whereas different uppercase letters indicate significant differences between biochar levels within the same nitrogen application rate at *p* < 0.05. The absence of letters indicates no significant difference (*p* > 0.05). Error bars represent standard errors of the mean (*n* = 5). B, biochar; N, nitrogen application rate; B × N, interaction between biochar and nitrogen application rate; ns, not significant. * *p* < 0.05; ** *p* < 0.01. The same notation applies to subsequent figures.

As shown in Figure 3, soil TC and TN concentrations exhibited a general pattern of initial increase followed by decrease with increasing N application rate, with the highest values observed under the B20N150 treatment. Compared with N0, the N150 treatment significantly increased soil TC and TN concentrations (*p* < 0.05). Under the same N fertilizer level, biochar amendment (B20) generally increased soil TC and TN, indicating that biochar addition facilitated soil carbon and nitrogen accumulation.

Soil TP concentration was also influenced by biochar and N fertilizer treatments. Among N levels, moderate N application tended to increase soil TP. At the N150 level, B20 significantly increased TP compared with B0 (*p* < 0.05). Overall, the B20N150 treatment exhibited the highest concentrations of TC, TN, and TP, suggesting that biochar combined with moderate N fertilization was most effective in enhancing total soil nutrient levels in the artificial grassland.

#### 2.2.2. Soil Ammonium and Nitrate

**Figure 4:**
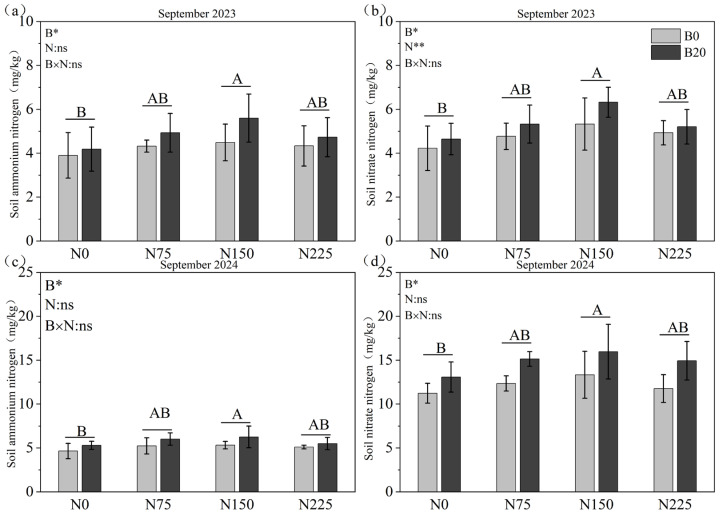
Effects of nitrogen rate and biochar addition on soil ammonium (NH_4_^+^–N) and nitrate (NO_3_^−^–N) concentrations. (**a**) Soil ammonium nitrogen in September 2023; (**b**) Soil nitrate nitrogen in September 2023; (**c**) Soil ammonium nitrogen in September 2024; (**d**) Soil nitrate nitrogen in September 2024 Note: N0, N75, N150, and N225 indicate nitrogen application rates of 0, 75, 150, and 225 kg N ha^−1^, respectively; B0 and B20 indicate biochar application rates of 0 and 20 t ha^−1^, respectively. Different uppercase letters indicate significant differences among nitrogen application rates under the same biochar level, whereas different uppercase letters indicate significant differences between biochar levels within the same nitrogen application rate at *p* < 0.05. The absence of letters indicates no significant difference (*p* > 0.05). Error bars represent standard errors of the mean (*n* = 5). B, biochar; N, nitrogen application rate; B × N, interaction between biochar and nitrogen application rate; ns, not significant. * *p* < 0.05; ** *p* < 0.01. The same notation applies to subsequent figures.

As presented in Figure 4, soil NH_4_^+^–N and NO_3_^−^–N concentrations responded markedly to biochar and N fertilizer treatments. Both parameters exhibited a pattern of initial increase followed by decrease with increasing N application rate, with the B20N150 treatment yielding the highest concentrations.

ANOVA results confirmed that both biochar and N fertilizer significantly affected soil NH_4_^+^–N and NO_3_^−^–N concentrations (*p* < 0.05). Under the same N level, B20 generally increased both NH_4_^+^–N and NO_3_^−^–N, indicating that biochar enhanced soil inorganic N availability. Compared with the unfertilized control, moderate N application significantly increased NH_4_^+^–N and NO_3_^−^–N; however, inorganic N concentrations did not continue to increase under the highest N rate (N225). Collectively, the B20N150 treatment exhibited the highest soil inorganic N concentrations, demonstrating that biochar combined with a moderate N rate was most effective in improving available N supply.

### 2.3. Effects of Nitrogen Rate and Biochar on Plant Community Diversity

**Figure 5:**
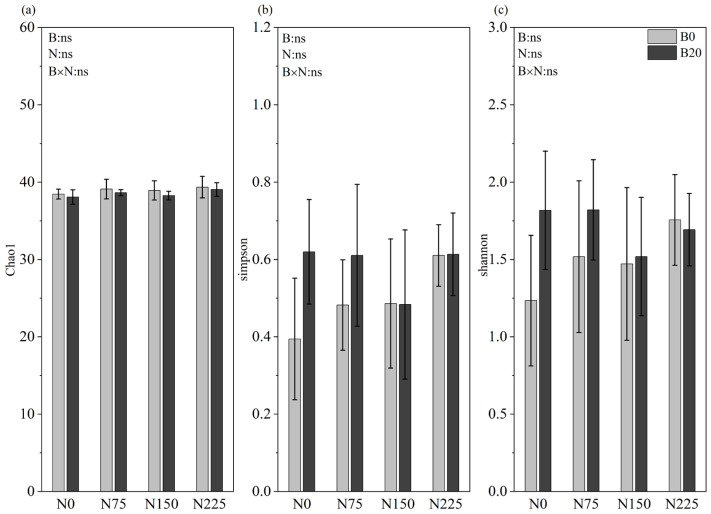
Effects of biochar and nitrogen rate addition on plant community diversity indices (Chao1, Simpson, and Shannon). (**a**) Chao1 index of plant communities; (**b**) Simpson index of plant communities; (**c**) Shannon index of plant communities Note: no pairwise comparisons reached significance (*p* > 0.05).

As shown in Figure 5, the Chao1, Simpson, and Shannon diversity indices did not differ significantly among treatments (*p* > 0.05). The Chao1 index exhibited minimal variation across treatments, indicating that the treatments had limited effects on species richness. In contrast, the Simpson and Shannon indices displayed some fluctuation among treatments; B20 tended to yield higher values than B0 under the N0 and N75 levels, suggesting a potential trend toward enhanced community diversity and evenness with biochar amendment under low-N conditions. However, pairwise comparisons confirmed that these trends did not reach statistical significance.

### 2.4. Responses of Hay Yield and Agronomic Nitrogen Use Efficiency to Nitrogen Rate and Biochar

#### 2.4.1. Hay Yield

**Figure 6:**
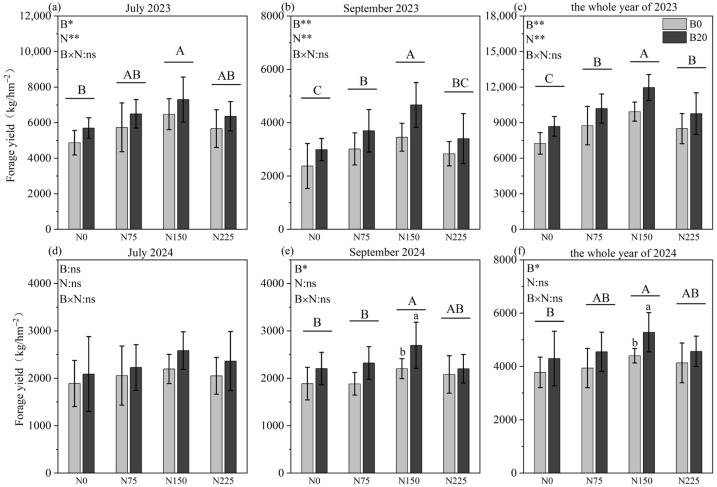
Effects of biochar and nitrogen application rate on hay dry matter yield in July, September, and the whole year of 2023 and 2024. (**a**) Forage yield of the first cutting in July 2023; (**b**) Forage yield of the second cutting in September 2023; (**c**) Annual total forage yield in 2023; (**d**) Forage yield of the first cutting in July 2024; (**e**) Forage yield of the second cutting in September 2024; (**f**) Annual total forage yield in 2024 Note: N0, N75, N150, and N225 indicate nitrogen application rates of 0, 75, 150, and 225 kg N ha^−1^, respectively; B0 and B20 indicate biochar application rates of 0 and 20 t ha^−1^, respectively. Different uppercase letters indicate significant differences among nitrogen application rates under the same biochar level, whereas different lowercase letters indicate significant differences between biochar levels within the same nitrogen application rate at *p* < 0.05. The absence of letters indicates no significant difference (*p* > 0.05). Error bars represent standard errors of the mean (*n* = 5). B, biochar; N, nitrogen application rate; B × N, interaction between biochar and nitrogen application rate; ns, not significant. * *p* < 0.05; ** *p* < 0.01. The same notation applies to subsequent figures.

As illustrated in Figure 6, hay yield was significantly increased under the N150 treatment compared with N0 (*p* < 0.05). Total annual hay yield was also significantly higher under N150 relative to N0 (*p* < 0.05). Under the N150 level, biochar application (B20) further increased total hay yield in 2024 (*p* < 0.05).

#### 2.4.2. Agronomic Nitrogen Use Efficiency

**Figure 7:**
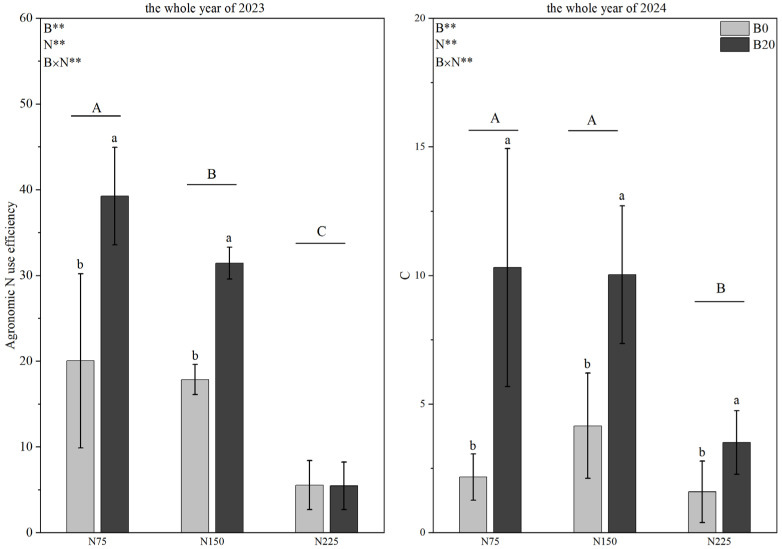
Effects of nitrogen rate and biochar addition on agronomic nitrogen use efficiency (NAUE). Note: N0, N75, N150, and N225 indicate nitrogen application rates of 0, 75, 150, and 225 kg N ha^−1^, respectively; B0 and B20 indicate biochar application rates of 0 and 20 t ha^−1^, respectively. Different uppercase letters indicate significant differences among nitrogen application rates under the same biochar level, whereas different lowercase letters indicate significant differences between biochar levels within the same nitrogen application rate at *p* < 0.05. The absence of letters indicates no significant difference (*p* > 0.05). Error bars represent standard errors of the mean (*n* = 5). B, biochar; N, nitrogen application rate; B × N, interaction between biochar and nitrogen application rate; ns, not significant. ** *p* < 0.01. The same notation applies to subsequent figures.

As shown in Figure 7, NAUE differed significantly among N fertilizer levels. Overall, NAUE was higher under low and moderate N rates and decreased under the highest N rate. Specifically, NAUE under N75 and N150 was significantly higher than under N15 (*p* < 0.05), indicating that reduced N application improved the yield return per unit of N input.

Under the same N level, biochar amendment generally increased NAUE, with some treatment comparisons reaching statistical significance (*p* < 0.05). Compared with sole N fertilization, the combined application of biochar and N fertilizer resulted in higher NAUE, demonstrating that biochar enhanced N fertilizer utilization efficiency. Among all treatments, B20N75 and B20N150 exhibited the highest NAUE values, with B20N150 achieving both high hay yield and high NAUE simultaneously.

### 2.5. Driving Factor Analysis of Agronomic Nitrogen Use Efficiency

#### 2.5.1. Linear Mixed-Effects Model Analysis

**Figure 8:**
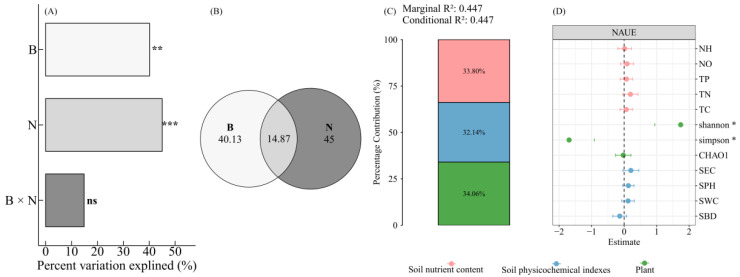
Linear mixed-effects model analysis of the relative contributions of biochar, nitrogen rate, and their interaction to agronomic nitrogen use efficiency variation. Note: (**A**) Variance contributions of biochar addition (B), nitrogen rate (N), and their interaction (B × N) to agronomic nitrogen use efficiency (NAUE) indicated by a linear mixed-effects model. *** *p* < 0.001; ** *p* < 0.01; ns: not significant. (**B**) Venn diagram showing unique and shared contributions of biochar (B) and nitrogen rate (N) to NAUE variance. B contributed 40.13%, N contributed 45%, and their interaction contributed 14.87%. (**C**) Stacked bar plot showing the percentage contributions of three factor types to NAUE: soil nutrient content (33.80%, red), soil physicochemical indices (32.14%, blue), and plant-related factors (34.06%, green). (**D**) Coefficients and significance levels of environmental variables in the LMM. Factors from soil nutrient content (red), soil physicochemical indices (blue), and plant-related indices (green) were analyzed. * 0.01 < *p* < 0.05; Abbreviations: NH: ammonium, NO: nitrate, TP: total phosphorus, TN: total nitrogen, TC: total carbon, SEC: soil electrical conductivity, SWC: soil water content, SBD: soil bulk density.

To further clarify the relative contributions of biochar, N fertilizer, and their interaction to NAUE variation, a linear mixed-effects model was employed. The results indicated that both biochar and N fertilizer significantly explained NAUE variation, accounting for 40.13% and 45.00% of the total variance, respectively. The interaction between biochar and N fertilizer explained 14.87% of the variance but did not reach statistical significance (Figure 8). These findings suggest that, under the conditions of this study, biochar and N fertilizer influenced NAUE primarily through independent rather than interactive effects.

Further grouped variable analysis revealed that plant-related indicators, soil nutrient indicators, and soil physicochemical indicators contributed comparably to NAUE variation, explaining 34.06%, 33.80%, and 32.14% of the variance, respectively. Both the marginal R^2^ and conditional R^2^ of the model were 0.447, indicating that the fixed effects explained approximately 44.7% of the total NAUE variation.

Individual effect estimates showed that among soil nutrient indicators, NH_4_^+^–N, NO_3_^−^–N, TP, TN, and TC all exhibited positive effects on NAUE, indicating that higher soil nutrient concentrations were closely associated with enhanced NAUE. Among plant-related indicators, the Shannon and Simpson indices exerted significant positive effects on NAUE (*p* < 0.05), whereas the Chao1 index had a weaker effect. Soil physicochemical indicators showed mixed directional effects on NAUE, suggesting that the influence of soil physical properties on NAUE is complex and warrants further investigation through structural equation modeling.

#### 2.5.2. Structural Equation Modeling

To disentangle the direct and indirect pathways through which biochar and N fertilizer affect NAUE, a structural equation model was constructed. The model demonstrated acceptable overall fit: χ^2^/df = 1.06, CFI = 0.988, TLI = 0.976, RMSEA = 0.039, and SRMR = 0.077. The model explained 35.9% of the variance in NAUE (R^2^ = 0.359).

**Figure 9:**
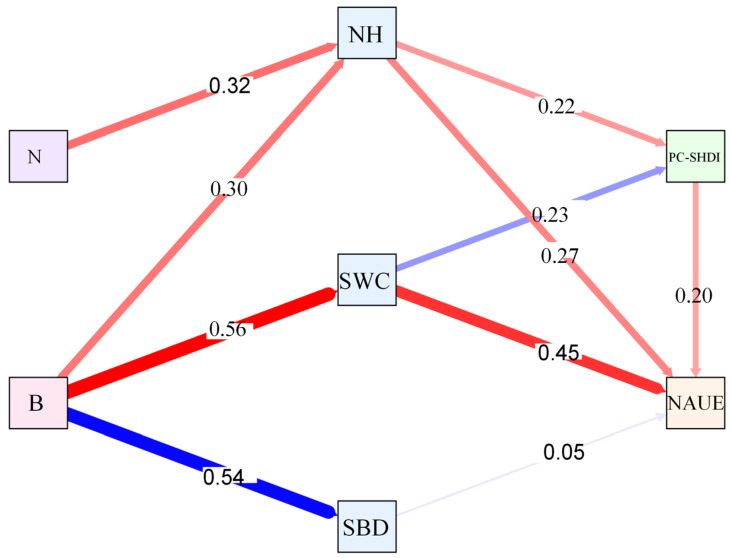
Structural equation model depicting the driving pathways of biochar and nitrogen addition on agronomic nitrogen use efficiency (NAUE). Note: Red arrows indicate positive relationships; blue arrows indicate negative relationships. Arrow thickness is proportional to the magnitude of the standardized path coefficient. Numbers adjacent to arrows represent standardized path coefficients. B, biochar addition; N, nitrogen rate addition; SBD, soil bulk density; SWC, soil water content; NH_4_^+^, ammonium; PC-SHDI, plant community Shannon diversity index; NAUE, agronomic nitrogen use efficiency.

The SEM results revealed that soil water content, NH_4_^+^–N, and plant community diversity all exerted positive direct effects on NAUE, with standardized path coefficients of 0.45, 0.27, and 0.20, respectively (Figure 9). Soil water content exhibited the strongest direct effect on NAUE, indicating that soil moisture status was a critical determinant of N use efficiency. The positive effect of NH_4_^+^–N on NAUE confirmed that enhanced available N supply facilitated improved N utilization. Plant community diversity also positively influenced NAUE, suggesting a functional linkage between community structure and N cycling processes.

Biochar exerted pronounced effects on soil bulk density, soil water content, and NH_4_^+^–N concentration, and influenced NAUE indirectly through these soil-mediated pathways. nitrogen rate primarily affected NAUE through its influence on soil NH_4_^+^–N concentration. Additionally, soil water content exhibited a negative effect on plant community diversity, suggesting that changes in soil moisture may influence plant community composition. Overall, the SEM demonstrated that biochar and N fertilizer primarily enhanced NAUE through the regulation of soil moisture, available N supply, and plant community diversity.

## 3. Discussion

### 3.1. Effects of Nitrogen Rate and Biochar on Soil Physicochemical Properties

Soil physicochemical properties provide an important basis for regulating productivity and nutrient use efficiency in artificial grasslands. In this study, biochar addition significantly reduced soil bulk density and increased soil water content, indicating a clear improvement in soil physical structure. This finding is consistent with previous studies showing that biochar, owing to its low density, porous structure, and large specific surface area, can improve soil porosity, reduce bulk density, and enhance soil water-holding capacity after incorporation into soil [29,30,31,32,33]. Particularly in arid and semiarid regions, soil water availability is often a key factor limiting forage growth and nitrogen uptake. Therefore, the increase in soil water content induced by biochar addition may improve the root growth environment and promote nutrient transport and absorption [34,35,36].

In the present study, the effects of biochar on soil bulk density and soil water content varied across nitrogen application rates, with the B20N150 treatment showing a relatively pronounced improvement. This suggests that the effect of biochar combined with nitrogen rate is not a simple additive response but is closely associated with nitrogen application rate [37]. Moderate nitrogen input can promote plant growth and root development, increasing root residues and organic matter inputs, thereby working together with biochar to improve soil structure. However, excessive nitrogen application may increase soil salinity or ionic concentrations, weakening soil structural stability and root activity. Thus, the combined application of biochar at the N150 level may be more conducive to creating a favorable soil physical environment.

In addition, biochar and nitrogen rate addition significantly affected soil electrical conductivity. Previous studies have reported that biochar contains ash and soluble mineral elements, which may temporarily increase soil soluble salt content after application [31,32]. Nitrogen rate hydrolysis and transformation can also increase the ionic concentration in the soil solution. The results of this study are generally consistent with these findings. However, because the soil in the study area is alkaline and the evaporation rate is high, whether long-term biochar and nitrogen rate application may lead to salt accumulation requires further investigation.

### 3.2. Effects of Nitrogen Rate and Biochar on Soil Nutrients

Soil carbon, nitrogen, and phosphorus contents directly determine soil fertility and the capacity of soil to supply nutrients for forage growth. The results of this study showed that the combined application of nitrogen rate and biochar increased soil total carbon, total nitrogen, total phosphorus, ammonium, and nitrate contents, with most nutrient indicators reaching relatively high levels under the B20N150 treatment. This finding is consistent with previous studies showing that biochar can enhance soil nutrient retention by directly supplying stable carbon sources, increasing nutrient adsorption, and reducing leaching losses [37,38].

The increase in soil total carbon content following biochar addition is mainly attributable to the stable carbon fractions contained in biochar, which are resistant to rapid decomposition and can therefore contribute to the soil carbon pool. In addition, by improving soil structure and water conditions, biochar may promote plant biomass production and root residue inputs, further enhancing soil organic carbon accumulation. With respect to soil nitrogen, nitrogen rate directly increases external nitrogen inputs, whereas biochar can adsorb ammonium and nitrate through its surface functional groups and porous structure, thereby reducing nitrogen volatilization and leaching losses and increasing soil available nitrogen content. Previous studies have also demonstrated that the combined application of biochar and nitrogen rate can significantly increase soil inorganic nitrogen content and delay nitrogen release processes [39,40], which is consistent with the results of the present study.

Notably, soil nutrient contents did not increase continuously with increasing nitrogen application rate in this study, but instead showed an initial increase followed by a decline, with the B20N150 treatment outperforming the B20N225 treatment. This indicates that excessive nitrogen application does not continuously improve soil nutrient availability; rather, it may reduce nutrient retention efficiency due to nitrate leaching, salt accumulation, or inhibition of microbial processes. Similar studies have reported that appropriate nitrogen application can promote soil nitrogen accumulation and plant uptake, whereas excessive nitrogen input reduces nitrogen use efficiency and increases the risk of environmental losses [41,42,43]. Therefore, the combined application of biochar with a moderate nitrogen rate may be more favorable for achieving a balance between soil nutrient accumulation and efficient nitrogen utilization.

### 3.3. Effects of Nitrogen Rate and Biochar on Hay Yield and Agronomic Nitrogen Use Efficiency

Nitrogen is a primary limiting nutrient for artificial grassland productivity. The present study demonstrated that the N150 treatment significantly increased hay yield, confirming that moderate N application can alleviate N limitation and promote forage growth and dry matter accumulation. This finding is consistent with previous reports that N fertilization enhances forage yield, plant height, and tiller number [6,7,8]. Nitrogen promotes chlorophyll and protein synthesis, enhances photosynthetic capacity, and thereby increases forage productivity.

However, when the N application rate was increased to N225, neither yield nor NAUE improved further, indicating the existence of a response threshold for N fertilization in artificial grasslands. Beyond this threshold, plant N uptake capacity may approach saturation, and surplus N is susceptible to loss through leaching, volatilization, or denitrification, resulting in diminishing marginal returns. Previous research has similarly documented that crop yield increments typically decline with increasing N rates, while N use efficiency decreases markedly [7,31]. Therefore, simply increasing N inputs is not an effective strategy for enhancing artificial grassland productivity.

Biochar amendment further improved NAUE, particularly under moderate N application rates. This enhancement may be attributed to the capacity of biochar to improve soil moisture and structure, thereby facilitating root nutrient acquisition, while simultaneously adsorbing and slowly releasing inorganic N to reduce N losses and increase the yield benefit per unit of N input. Previous studies have demonstrated that biochar combined with N fertilizer can improve fertilizer use efficiency and crop yield while reducing N loss risks [13,18], consistent with the present findings.

The linear mixed-effects model further revealed that biochar and N fertilizer each exerted strong independent explanatory effects on NAUE, suggesting that they influence N utilization through distinct pathways. nitrogen rate primarily enhances NAUE by increasing available N supply to promote forage growth, whereas biochar primarily improves NAUE by ameliorating the soil physical environment and enhancing nutrient retention capacity. The SEM results corroborated this interpretation, identifying SWC and NH_4_^+^–N as the most important direct drivers of NAUE, thereby establishing the pathway of “moisture improvement → N retention → yield formation” as a key mechanism through which biochar and N fertilizer co-application enhances N use efficiency.

Additionally, plant community diversity exerted a positive effect on NAUE. Higher plant diversity facilitates complementary resource use among species through differentiation in root distribution, temporal patterns of nutrient uptake, and resource acquisition strategies, thereby enhancing the overall capacity of the community to intercept and utilize N. This finding is consistent with the niche complementarity hypothesis in biodiversity–ecosystem functioning theory, which posits that more diverse plant communities generally exhibit higher resource use efficiency and greater production stability. Therefore, in the fertilization management of artificial grasslands, maintaining an appropriate plant community structure should be considered alongside optimizing soil nutrient supply.

### 3.4. Optimal Treatment Combination and Practical Implications

Integrating the results for soil physicochemical properties, soil nutrients, hay yield, and NAUE, the present study identifies the B20N150 treatment—20 t·ha^−1^ biochar combined with 150 kg·ha^−1^ N fertilizer—as the optimal management combination under the experimental conditions. This treatment significantly improved soil bulk density and water content, increased soil TC, TN, TP, and available N concentrations, and simultaneously enhanced hay yield and NAUE. Compared with the conventional high-N treatment (N225), the N150 level reduced N fertilizer input by approximately 33.3% while maintaining comparable or higher yield, demonstrating the N-reduction and efficiency-enhancement potential of biochar co-application.

This finding aligns with current national priorities for green agricultural development and chemical fertilizer reduction. For artificial grasslands in arid and semiarid regions, insufficient soil moisture, declining fertility, and low N use efficiency are primary constraints on production. Biochar combined with moderate N fertilization can address these constraints through improved soil structure, enhanced water and nutrient retention, and increased nutrient availability, thereby achieving synergistic benefits of soil amelioration and forage yield enhancement.

Nevertheless, several limitations of the present study should be acknowledged. First, the experimental duration was limited to two years, and the long-term aging effects of biochar in soil and its sustained efficacy require verification through extended monitoring. Second, N loss pathways—including ammonia volatilization, nitrate leaching, and N_2_O emissions—were not quantified, precluding a comprehensive environmental impact assessment. Third, only a single biochar application rate was tested; future studies should include multiple biochar rates and soil types to determine optimal application thresholds and regional applicability.

In summary, biochar combined with moderate N fertilization can enhance artificial grassland hay yield and NAUE by improving the soil physical environment, increasing soil nutrient retention capacity, and promoting complementary resource utilization within the plant community. The B20N150 treatment demonstrated superior performance under the conditions of this study and may serve as a reference for N-reduction strategies and soil quality improvement in artificial grasslands of the arid and semiarid regions of Xinjiang.

## 4. Materials and Methods

### 4.1. Study Site Description

The experiment was conducted at the Grassland Science Experimental Station of Xinjiang Agricultural University, located in Xiejia’gou Village, Urumqi County, Xinjiang, China (43°52′ N, 87°09′ E; elevation 1630 m a.s.l.). The site is situated on the mid-to-low mountain belt of the northern Tianshan Mountains, characterized by gently undulating foothill steppe topography. The regional climate is classified as continental temperate, with a mean annual temperature of 3.3 °C, mean annual precipitation of approximately 300 mm (concentrated primarily during the summer months), and annual potential evaporation of 1100–1300 mm, yielding a pronounced aridity index (E_p_/P ≈ 3.7–4.3). The frost-free period spans 120–140 days per year. The soil is classified as mountain chestnut soil (Calcic Kastanozem according to the FAO–WRB classification system). Baseline soil physicochemical properties measured prior to treatment establishment are presented in Table 2, with no significant differences detected among plots.

The experimental field was established as an artificial grassland in 2013 through mechanical tillage followed by mixed sowing of *Onobrychis viciaefolia Scop.* (sainfoin), *Medicago sativa L.* (alfalfa), and *Elytrigia repens* (L.) Desv. ex Nevski (quackgrass). Since establishment, the site has been subjected to annual mowing for hay harvest without any additional fertilization, irrigation, grazing, or other management interventions, thus representing a typical long-term nutrient-depleting artificial grassland in the arid–semiarid region of northern Xinjiang.

### 4.2. Experimental Materials

The biochar used in this study was produced from maize (*Zea mays* L.) straw by slow pyrolysis at temperatures ranging from 300 to 700 °C and was supplied by Henan Jiahe Water Purification Materials Co., Ltd. (Zhengzhou, Henan, China). The biochar was ground to pass through a 200-mesh sieve (particle diameter < 75 μm) and exhibited the following properties: pH value of 9.36, specific surface area of 603 m^2^·g^−1^, total carbon content of 70.2%, total nitrogen content of 1.61%, cellulose content of 16.85%, and ash content of 12.95%.

### 4.3. Experimental Design

The experiment was initiated in April 2023 on the aforementioned artificial grassland established in 2013. A two-factor completely randomized block design was adopted, with biochar (B) and nitrogen rate (N) as the two experimental factors. Two biochar levels were established: B0 (0 t·ha^−1^, control) and B20 (20 t·ha^−1^, a commonly recommended application rate for cropland soils). Four N fertilizer levels were established based on an equal-gradient reduction from the conventional N application rate for artificial grasslands in the region (225 kg·ha^−1^): N0 (0 kg·ha^−1^; 100% reduction), N75 (75 kg·ha^−1^; 66.7% reduction), N150 (150 kg·ha^−1^; 33.3% reduction), and N225 (225 kg·ha^−1^; 0% reduction, i.e., the conventional rate).The nitrogen rate used was urea (N ≥ 46%). The factorial combination of two biochar levels and four N levels yielded eight treatments: B0N0, B0N75, B0N150, B0N225, B20N0, B20N75, B20N150, and B20N225 (Table 3). Each treatment was replicated five times, resulting in a total of 40 experimental plots. Individual plot dimensions were 2 m × 4 m (8 m^2^), with 1 m buffer strips between adjacent plots to prevent lateral nutrient migration.

In late May 2023, biochar was surface-broadcast onto the designated plots and thoroughly incorporated into the 0–20 cm topsoil layer using manual tillage. A light water spray was applied immediately after incorporation to facilitate biochar adhesion to soil particles and minimize wind-induced losses. Biochar was applied only once at the onset of the experiment and was not reapplied during the two-year study period. nitrogen rate (urea) was dissolved in a small volume of water and uniformly sprayed onto the plot surface at three equal split applications per year (late May, late July, and late September) during both 2023 and 2024.

### 4.4. Sampling and Analytical Methods

#### 4.4.1. Plant Sampling and Measurements

Hay yield determination. ** In September of both 2023 and 2024, aboveground biomass in each plot was harvested using a lawn mower at a stubble height of 8 cm. Two harvests were conducted annually (July and September). The fresh weight of the total harvested biomass per plot was recorded as the standing fresh hay yield. A random subsample of 200 g fresh biomass was collected from each plot, transported to the laboratory, and oven-dried at 65 °C for 72 h to constant weight. The fresh-to-dry weight ratio was calculated and used to convert fresh hay yield to dry matter yield, which was subsequently used for NAUE calculations.

Plant community survey. Vegetation surveys were conducted during the peak flowering stage of the forage growing season. Within each treatment plot, one 1 m × 1 m quadrat was randomly established. All plant species within the quadrat were identified, and the following parameters were recorded for each species: individual count (density), canopy cover, plant height, and aboveground biomass. Aboveground plant samples were sorted by species, transported to the laboratory, and oven-dried at 65 °C to constant weight for dry matter determination.

Plant community diversity indices. Community diversity was characterized using the Shannon–Wiener index (H′), Simpson index (D), and Chao1 richness estimator, calculated as follows:

H′ = −Σ(P_i_ lnP_i_)

D = 1 − ΣP_i_^2^

Chao1 = Sobs + F_1_^2^/2F_2_

where Sobs is the observed number of species, F_1_ is the number of singleton species (species observed exactly once), and F_2_ is the number of doubleton species (species observed exactly twice). When F_2_ = 0, a bias-corrected formula was applied:

Chao1 = Sobs + F_1_(F_1_ − 1)/[2(F_2_ + 1)]

Higher values of H′ indicate greater species diversity; higher values of D indicate greater evenness and diversity; and higher Chao1 values indicate greater estimated species richness.

#### 4.4.2. Soil Sampling and Analytical Methods

Soil samples were collected using a soil auger (5 cm diameter) from the 0–10 cm topsoil layer within each quadrat after aboveground vegetation had been removed. Two soil cores were extracted per plot following a diagonal sampling pattern and combined into a single composite sample per plot. Samples were placed in labeled self-sealing polyethylene bags and stored in insulated containers for transport to the laboratory.

Upon return to the laboratory, samples were passed through a 2 mm mesh sieve to remove plant roots and coarse debris. Root fragments retained on the sieve were cleaned, oven-dried at 65 °C for 72 h, and weighed separately. Each sieved soil sample was divided into three subsamples: one was stored at 4 °C for fresh soil analyses (NH_4_^+^–N and NO_3_^−^–N), and the remaining two were air-dried in a cool, ventilated area for subsequent physicochemical analyses.

Soil analytical methods followed standardized protocols described in Soil Agrochemical Analysis [27]. Specific methods were as follows: soil pH was determined using a pH meter (water-to-soil ratio of 2.5:1); electrical conductivity (EC) was measured using a conductivity meter (water-to-soil ratio of 2.5:1); soil water content (SWC) was determined gravimetrically by oven-drying at 105 °C for 24 h; soil bulk density (SBD) was measured using the core-ring method; total carbon (TC) was determined by the potassium dichromate–external heating method; total nitrogen (TN) was measured by the Kjeldahl method; total phosphorus (TP) was determined by alkali fusion followed by molybdenum–antimony colorimetry; and NH_4_^+^–N and NO_3_^−^–N concentrations in fresh soil samples were quantified using a continuous flow analyzer (AA3, SEAL Analytical, Norderstedt, Germany).

### 4.5. Data Analysis and Statistical Methods

Raw data were organized and pre-processed using Microsoft Excel (version 2019). Statistical analyses were performed using SPSS 25.0 (IBM Corp., Armonk, NY, USA). The effects of biochar, N fertilizer, year, and their interactions on soil physicochemical properties were assessed using repeated-measures analysis of variance (RM-ANOVA). Differences among treatments were evaluated using Duncan’s multiple range test and paired *t*-tests at a significance level of *p* < 0.05. Pearson correlation analysis was conducted using Origin 2022 (OriginLab Corp., Northampton, MA, USA). Figures were generated using SigmaPlot 14.0 (Systat Software Inc., San Jose, CA, USA).

To quantify the relative contributions of biochar, N fertilizer, and their interaction to NAUE variation, a linear mixed-effects model analysis was performed using the “glmm.hp” package in R. Structural equation modeling (SEM) was conducted using the “lavaan” package in R (version 4.3.0) with maximum likelihood (ML) estimation and a maximum iteration limit of 2000. Model fit was evaluated using the following indices: chi-square to degrees of freedom ratio (χ^2^/df), comparative fit index (CFI), Tucker–Lewis index (TLI), root mean square error of approximation (RMSEA), and standardized root mean square residual (SRMR). The coefficient of determination (R^2^) was calculated for each endogenous variable to assess the explanatory power of the model.

Agronomic nitrogen use efficiency (NAUE; kg·kg^−1^) was calculated as:

NAUE = (Y_N_−Y_0_)/F_N_

where Y__N_ is the hay yield (kg·ha^−1^) of the N-fertilized treatment, Y_0_ is the hay yield of the unfertilized control (N0) under the corresponding biochar level, and F__N_ is the amount of N fertilizer applied (kg·ha^−1^).

## 5. Conclusions

The present study demonstrated that the combined application of biochar and nitrogen rate significantly improved the soil environment of artificial grasslands and enhanced agronomic nitrogen use efficiency. Biochar amendment significantly reduced soil bulk density, increased soil water content, and enhanced soil water-holding capacity, while simultaneously increasing soil total carbon, total nitrogen, total phosphorus, ammonium, and nitrate concentrations, thereby promoting soil nutrient accumulation. Compared with sole nitrogen fertilization, the co-application of biochar with moderate nitrogen rate was more effective in improving soil physical structure and Nutrient supply.

Nitrogen rate application significantly influenced hay yield and NAUE; however, these effects did not increase continuously with increasing N rate. The moderate N level (150 kg·ha^−1^, N150) significantly increased hay yield, whereas the high N treatment (N225) did not further enhance yield and reduced NAUE. Biochar amendment improved NAUE to a certain extent, with the B20N150 treatment (20 t·ha^−1^ biochar combined with 150 kg·ha^−1^ N fertilizer) achieving simultaneously high hay yield, favorable soil nutrient status, and high NAUE, representing the optimal treatment combination under the conditions of this study.

Plant community diversity indices did not respond significantly to biochar or N fertilizer treatments, indicating that these amendments did not markedly alter plant community structure within the short-term experimental period. However, under low-N conditions, biochar-amended treatments exhibited slightly higher Simpson and Shannon indices, suggesting a potential role of biochar in maintaining or improving community diversity.

Linear mixed-effects modeling and structural equation modeling further revealed that biochar and N fertilizer primarily influenced NAUE through the improvement of soil moisture conditions, enhancement of available N supply, and regulation of plant community diversity. Soil water content and ammonium were identified as the key driving factors for NAUE enhancement. In conclusion, the combined application of biochar with moderate N fertilizer represents an effective management strategy for N reduction, efficiency enhancement, and soil quality improvement in artificial grasslands of the arid and semiarid regions of Xinjiang.

## Figures and Tables

**Table 1:** Repeated-measures ANOVA results for the effects of biochar (B), nitrogen rate (N), year, and their interactions on soil physicochemical properties (2023–2024).

	B		N		B × N		Year		B × Year		N × Year		B × N × Year	
	F	P	F	P	F	P	F	P	F	P	F	P	F	P
SBD (g/cm^3^)	28.40	**	0.26	0.85	0.26	0.85	55.50	**	2.57	0.12	0.71	0.55	0.13	0.94
SWC (%)	40.54	**	7.31	**	0.46	0.71	11.93	**	0.01	0.93	0.36	0.78	0.94	0.43

Note: SBD, soil bulk density; SWC, soil water content. ** indicate significance at *p* < 0.05 and *p* < 0.01, respectively.

**Table 2:** Baseline soil physicochemical properties prior to treatment establishment. Soil water content was measured gravimetrically from soil samples collected before treatment application.

Soil pH	Electric Conductivity (mS/cm^3^)	Bulk Density (g/cm^3^)	Water Content (%)	Total Carbon (g/kg)	Total Nitrogen (g/kg)	Total Phosphorus (g/kg)
8.54 ± 0.9	125.48 ± 30.35	1.51 ± 0.07	9.63 ± 0.93	18.8 ± 2.01	2.45 ± 0.33	0.36 ± 0.2

**Table 3:** Experimental treatments and corresponding fertilizer application rates.

Treatment	Biochar (t/hm^2^)	Nitrogen Rate (kg/hm^2^)
B0N0	0	0
B0N75	0	75
B0N150	0	150
B0N225	0	225
B20N0	20	0
B20N75	20	75
B20N150	20	150
B20N225	20	225

## Data Availability

The data supporting the findings of this study are available from the corresponding author upon reasonable request. The data are not publicly available due to privacy restriction.

## Abbreviations

The following abbreviations are used in this manuscript:

B	Biochar application
B × N	Interaction between biochar and nitrogen rate
B0	Biochar application rate of 0 t·ha^−1^
B0N0	0 t·ha^−1^ biochar with 0 kg·ha^−1^ nitrogen rate
B0N75	0 t·ha^−1^ biochar with 75 kg·ha^−1^ nitrogen rate
B0N150	0 t·ha^−1^ biochar with 150 kg·ha^−1^ nitrogen rate
B0N225	0 t·ha^−1^ biochar with 225 kg·ha^−1^ nitrogen rate
B20N0	20 t·ha^−1^ biochar with 0 kg·ha^−1^ nitrogen rate
B20N75	20 t·ha^−1^ biochar with 75 kg·ha^−1^ nitrogen rate
B20N150	20 t·ha^−1^ biochar with 150 kg·ha^−1^ nitrogen rate
B20N225	20 t·ha^−1^ biochar with 225 kg·ha^−1^ nitrogen rate
CFI	Comparative fit index
D	Simpson diversity index
EC	Electrical conductivity
H′	Shannon–Wiener diversity index
IV	Importance value
ML	Maximum likelihood estimation
N	Nitrogen rate application
N0	Nitrogen rate application rate of 0 kg·ha^−1^
N75	Nitrogen rate application rate of 75 kg·ha^−1^
N150	Nitrogen rate application rate of 150 kg·ha^−1^
N2250	Nitrogen rate application rate of 225 kg·ha^−1^
NAUE	Agronomic nitrogen use efficiency
NH_4_^+^–N	Ammonium
NO_3_^−^–N	Nitrate
*p*	Probability value
PCA	Principal component analysis
PC-SHDI	Plant community Shannon diversity index
R^2^	Coefficient of determination
RB	Relative biomass
RC	Relative cover
RD	Relative density
RM-ANOVA	Repeated-measures analysis of variance
RMSEA	Root mean square error of approximation
SBD	Soil bulk density
SEM	Structural equation modeling
SRMR	Standardized root mean square residual
SWC	Soil water content
TC	Total carbon
TLI	Tucker–Lewis index
TN	Total nitrogen
χ^2^/df	Chi-square to degrees-of-freedom ratio
